# Social Attention in the Two Species of Pan: Bonobos Make More Eye Contact than Chimpanzees

**DOI:** 10.1371/journal.pone.0129684

**Published:** 2015-06-15

**Authors:** Fumihiro Kano, Satoshi Hirata, Josep Call

**Affiliations:** 1 Kumamoto Sanctuary, Wildlife Research Center, Kyoto University, Kumamoto, Uki, Japan; 2 Primate Research Institute, Kyoto University, Inuyama, Japan; 3 Department of Developmental and Comparative Psychology, Max-Planck Institute for Evolutionary Anthropology, Leipzig, Germany; 4 Japan Society for Promotion of Science, Tokyo, Japan; 5 School of Psychology and Neuroscience, University of St Andrews, St Andrews, United Kingdom; University of Florence, ITALY

## Abstract

Humans’ two closest primate living relatives, bonobos and chimpanzees, differ behaviorally, cognitively, and emotionally in several ways despite their general similarities. While bonobos show more affiliative behaviors towards conspecifics, chimpanzees display more overt and severe aggression against conspecifics. From a cognitive standpoint, bonobos perform better in social coordination, gaze-following and food-related cooperation, while chimpanzees excel in tasks requiring extractive foraging skills. We hypothesized that attention and motivation play an important role in shaping the species differences in behavior, cognition, and emotion. Thus, we predicted that bonobos would pay more attention to the other individuals’ face and eyes, as those are related to social affiliation and social coordination, while chimpanzees would pay more attention to the action target objects, as they are related to foraging. Using eye-tracking we examined the bonobos’ and chimpanzees’ spontaneous scanning of pictures that included eyes, mouth, face, genitals, and action target objects of conspecifics. Although bonobos and chimpanzees viewed those elements overall similarly, bonobos viewed the face and eyes longer than chimpanzees, whereas chimpanzees viewed the other elements, the mouth, action target objects and genitals, longer than bonobos. In a discriminant analysis, the individual variation in viewing patterns robustly predicted the species of individuals, thus clearly demonstrating species-specific viewing patterns. We suggest that such attentional and motivational differences between bonobos and chimpanzees could have partly contributed to shaping the species-specific behaviors, cognition, and emotion of these species, even in a relatively short period of evolutionary time.

## Introduction

Despite their general similarities and close phylogenetic relationship, bonobos and chimpanzees show some important differences, particularly in their patterns of aggression and affiliation. While chimpanzees often engage in aggressive displays and severe aggression which occasionally involves the killing of conspecifics, bonobos rarely exhibit such overt aggressive behaviors [1–3]. Moreover, bonobos exhibit a larger repertoire of affiliative behaviors towards conspecifics compared to chimpanzees including non-conceptive sexual behaviors, frequent play among adults, and non-aggressive encounters with strangers [1, 4–6]. It has been hypothesized that bonobo behavior has evolved in part as a response to the relaxation of intra-group competition and selection against male aggression [1, 3].

In humans, eye contact is related to the level of affiliation among individuals, and thus it reflects an individual’s temperament and the interpersonal relationships [7, 8]. People facing each other tend to reach an equilibrium in both physical distance and eye contact that depends on their affiliative motivation and the approach-avoidance conflict; people with a more relaxed relationship with their partners and with a higher need for affiliation show a closer physical distance and an increased level of eye contact. The level of eye contact is also modulated by social parameters such as cultural background and clinical condition [8–10]. If eye contact is modulated by the same principles in bonobos and chimpanzees as in humans, it is predicted that bonobos, the species with an increased affiliative motivation, would make more eye contact than chimpanzees.

Non-human primates have much in common with humans in terms of the pattern and function of eye contact [11]. From an early age, humans and chimpanzees preferentially orient to faces looking at vs. looking away from observers [12, 13]. Visual search experiments have shown that humans and chimpanzees are able to detect such direct gaze faster than averted gaze [14, 15]. Eye-tracking experiments have shown that humans and several species of nonhuman primates predominantly fixate eyes among facial features [16–21]. Observational studies have found that, although prolonged eye contact is not commonly observed among adults in nonhuman primates as it serves as a threat to the conspecifics [11], eye contact plays an important role in affiliative contexts. Mothers and infants in macaques and chimpanzees exchange frequent eye contact and facial expressions [22, 23]. When chimpanzees and gorillas make an attempt to reconcile with conspecifics after fighting, they first establish eye contact before approaching their counterparts [24, 25].

Only few studies examined the individual and species variation of eye contact in non-human primates. One study examined the eye-contact frequency of six monkey species in response to an experimenter approaching the subjects [26]. Rhesus macaques exhibited the lowest frequencies of eye contact. Also, young individuals, especially females, exhibited more eye-contact than adult individuals. Using eye-tracking, two other studies examined eye-fixation in great apes and humans in response to conspecific faces [16, 17]. Chimpanzees, gorillas, and orangutans extensively viewed the eyes of conspecific images as did humans. However, humans exhibited a more prolonged viewing of the eyes compared to great apes. These results are largely consistent with the idea that species variation in eye contact is related to the species variation in affiliative motivation; those species with more despotic and less tolerant social systems tended to show a lower level of eye contact than those with more egalitarian and tolerant social systems. However, comparing chimpanzees and bonobos, by virtue of their close phylogenetic distance and the differences in affiliation and aggression, offers a more refined test of the hypothesis that affiliation and eye contact are positively associated in primate species.

Another important aspect of eye contact is that it plays a foundational role in the development of behavior and cognition in humans [27]. Humans orient to others’ eyes from birth [12], and eye contact facilitates the brain network related to social communication [27]. Preverbal infants later diagnosed with autistic spectrum disorder (ASD) show a decreased level of eye contact compared to typically-developing (TD) infants as early as 2–6 months of age [28]. The decreased level of eye contact is correlated with the increased level of ASD [10]. Importantly, despite the lack of social skills, some people with ASD show outstanding cognitive abilities in some physical domains [29] (“empathizing” and “systemizing” in a related theory; [30]), suggesting a degree of trade-off between socio-emotional and physical cognition in human development. We thus need to consider the possibility that, as in humans, attentional and motivational biases constitute a proximal cause for cognitive differences also in non-human primates.

Herrmann et al. [31] conducted a broad range of cognitive tests covering both social and physical domains in bonobos and chimpanzees. Bonobos outperformed chimpanzees in tasks related to theory-of-mind, especially gaze-following [32], while chimpanzees outperformed bonobos in tool-using and physical causality tasks. Consistent with these results, previous studies reported that bonobos cooperated better with conspecifics in obtaining food due to their higher tolerance levels compared to chimpanzees [33]. Other studies have reported that chimpanzees outperform bonobos in spatial-memory and wait longer for larger foods in temporal-discounting task [34, 35]. In addition, chimpanzees are well-known for their complex extractive-foraging and tool-using techniques, and the social transmission of those techniques in both captive and wild populations [36, 37], while extractive-foraging is relatively infrequent and tool-using in feeding contexts is virtually inexistent in wild bonobos [38–40].

It is noteworthy that such potential cognitive differences between bonobos and chimpanzees may depend on the attentional and motivational differences of the two species, rather than on their cognitive abilities per se. On the one hand, when tested in the laboratory, some bonobo participants showed equivalent or even superior abilities in extractive-foraging and tool-using techniques compared to chimpanzees [41–44]. Chimpanzees possess a remarkable ability to solve a variety of theory-of-mind tasks, especially in competitive contexts [45]. On the other hand, a recent study has reported that object-play among juveniles was more frequent in chimpanzees than bonobos, while social-play was equally frequent in the two species of juveniles [46]but another study reported that social-play among adults was more frequent in bonobos than chimpanzees [4].

Currently lacking is the experimental comparison of the two species’ “interest”; how bonobos and chimpanzees spontaneously attend to social stimuli without any task demands. A recent study using the eye-tracking method found that the degree of eye-fixation while viewing naturalistic images could reliably predict the degree of socio-emotional development in human infants [28]. Using a similar approach, in this study, we aimed to elucidate the differences between bonobos and chimpanzees in social attention.

Specifically, we examined the eye movements of bonobos and chimpanzees when they freely viewed naturalistic pictures of both bonobos and chimpanzees (i.e., the same set of stimuli). The stimuli included elements such as the face, eyes, mouth, and action target objects of other individuals. Our prediction was that, when looking at the other individuals, bonobos would pay more attention to the parts related to socio-emotional skills such as face and eyes, and chimpanzees would pay more attention to the parts related to extractive foraging such as the action target object.

The first set of stimuli depicted full faces of apes including the eyes and mouth, and the second set of pictures depicted the full bodies of the apes including the face, and action target objects (e.g. tools, foods, toys). We additionally included stimuli depicting the ano-genital area of individuals in the full-body pictures because it is known to strongly attract primates’ attention [47], thus serving as a control distractor for the other elements.

## Methods

### Participants

Fourteen bonobos (*Pan paniscus*) and 20 chimpanzees (*Pan troglodytes*) participated in this study (Table 1). Each species lived with their conspecifics and had only visual access to the other species. The sex and age of participants were balanced as much as possible between species. Six bonobos and 6 chimpanzees lived at Kumamoto Sanctuary (KS), Japan, and 8 bonobos and 14 chimpanzees lived at the Wolfgang Köhler Primate Research Center (WKPRC), Germany. These apes previously participated in the eye-tracking experiments with similar methods [21, 32, 48, 49], yet they were never explicitly trained to fixate on certain stimuli or change their viewing patterns.

**Table 1:** The participant information and the viewing times (in millisecond) to each AOI.

Species	Facility	Sex	Age	Rearing H.	Name	Eye (ms)	Mouth (ms)	Face (ms)	Genital (ms)	Target (ms)	Missclassified	Discriminant Score
Bonobo	KS	M	10	Nursery	Vijay	1368	302	1232	178	455		-3.20
	MPI	F	21	Mother	Ulindi	972	489	1034	400	292		-2.40
	MPI	F	9	Mother	Luiza	1158	659	1269	450	478		-2.33
	KS	F	32	Mother	Lenor	681	678	799	256	175		-2.17
	KS	F	23	Nursery	Ikera	1461	356	1006	562	379		-1.82
	KS	F	42	Nursery	Loise	1093	398	807	226	408		-1.61
	MPI	M	18	Nursery	Kuno	1142	508	827	391	436		-1.14
	MPI	M	24	Mother	Jasongo	965	694	1078	532	615		-0.80
	MPI	F	6	Mother	Fimi	831	547	825	503	451		-0.68
	KS	M	19	Mother	Junior	821	457	748	758	295		-0.50
	MPI	M	32	Nursery	Joey	1253	857	815	479	553		-0.27
	MPI	M	5	Mother	Loto	519	1106	922	550	563	*	-0.03
	MPI	F	17	Mother	Yasa	924	522	896	596	669	*	0.14
	KS	F	25	Nursery	Loleta	163	343	456	570	316	*	0.37
Chimpanzee	MPI	F	37	Nursery	Ulla	1455	471	1098	739	474	*	-1.28
	MPI	F	39	Nursery	Riet	1247	675	863	811	370	*	-0.51
	KS	M	18	Mother	Jamba	249	152	663	601	392		-0.11
	MPI	M	9	Mother	Kofi	361	923	492	318	403		0.25
	MPI	M	13	Nursery	Alex	816	1027	1004	340	901		0.43
	MPI	F	9	Mother	Kara	406	752	565	251	606		0.57
	KS	F	5	Mother	Iroha	679	413	602	673	474		0.59
	MPI	M	13	Mother	Lome	461	878	650	560	506		0.62
	MPI	F	21	Mother	Jahaga	978	481	722	830	523		0.66
	MPI	F	21	Mother	Sandra	751	1232	896	867	535		0.66
	MPI	F	21	Mother	Getrudia	417	394	337	375	421		0.71
	MPI	M	9	Mother	Lobo	543	897	735	555	611		0.72
	KS	F	15	Mother	Misaki	581	272	645	729	557		0.87
	MPI	M	39	Nursery	Robert	546	987	634	818	490		1.24
	KS	F	17	Nursery	Mizuki	764	370	699	470	859		1.31
	KS	F	5	Nursery	Hatsuka	466	546	544	496	664		1.32
	MPI	M	5	Mother	Bangolo	420	941	585	284	771		1.35
	MPI	F	38	Nursery	Fraukje	819	968	616	811	657		1.87
	MPI	F	21	Mother	Fifi	379	458	324	530	664		2.17
	KS	F	8	Mother	Natsuki	883	725	620	1097	809		2.99

The participants misclassified by the discriminant analysis were marked by the asterisks. The participants were arranged in the order of discriminant scores within each species.

### Ethics statement

Animal husbandry and research complied with the international standards and the local guidelines which are strictly adhered to the national laws of Japan or Germany. See S1 File (ethics statement) for the details.

### Apparatus

The eye movements of apes were noninvasively recorded with an infrared Tobii eye tracker (60 Hz; Tobii Technology AB, Stockholm, Sweden). Stimulus pictures were presented using Tobii Studio software on a 22-in. LCD monitor (1,366×768 pixels) at a 70-cm viewing distance (1 degree of gaze angle corresponded to approximately 1.2 cm on the monitor). In order to keep their heads relatively still, we adopted two different methods depending on the opportunities available at each facility. As KS bonobos and WKPRC bonobos/chimpanzees were separated from the experimenter and eye tracker by a transparent acrylic panel, we attached a nozzle and tube that dripped grape juice to the acrylic panel and let them suck the nozzle during recording (see Figure A in S1 File). As KS chimpanzees were able to stay with an experimenter in the testing room, one of the experimenters stayed inside the room, sat beside the subjects, and lightly held the chin. The other experimenter and the eye tracker stayed outside the room and recorded the subjects’ eyes through the transparent acrylic panel. These methodological differences, however, did not directly influence the results, as we see below (see Table 1). The apes did not receive any explicit training for viewing the stimuli.

### Calibration

Two-point automated calibration was conducted by presenting a small object on each reference point. Relatively small numbers of reference points were used in this study because apes tended to view those reference points only briefly. However, we manually checked accuracy at five points after the initial calibration and repeated the calibration if necessary. With this procedure, a validation session with 19 apes obtained accuracy comparable to that obtained with human participants (the positional error was, on average, 0.5–0.7 degree on the screen). For the details, see [48].

### Stimuli and procedures

Stimuli were 45 pictures of bonobos and 45 pictures of chimpanzees (total 90) from WKPRC individuals (thus these pictures included the individuals familiar to WKPRC individuals but not to KS individuals). There were 30 full-face, forward-facing pictures of apes (15 bonobo and 15 chimpanzee). The remaining 60 pictures were full-body pictures (30 bonobo and 30 chimpanzee) which included the faces (in all pictures) and the ano-genital areas (in 36 pictures) and the objects that were handled by the model apes (e.g. food, tools, toys; in 37 pictures; 13 pictures included both objects and genitals). Each picture was 1.0–1.5 in aspect ratio, and was presented at the maximum size on the screen (22-inch, 16:9, 48.7×27.4 cm, 1366×768 pixel). Each picture was presented to a subject for 3 seconds (trial), and each day (session) presented 8 to 10 pictures of either species consecutively, as a slide show (total 10 days). The presentation order of pictures was randomized for each subject. A session was initiated when we confirmed that the error value was less than 1.5 degree around the center of the screen. In case the eye-tracking signals was severely lost (e.g. apes left the front of the monitor during the recording), which occurred in 6.1% of all sessions, the same session was repeated on the next day.

### Data analysis

**Fig 1:**
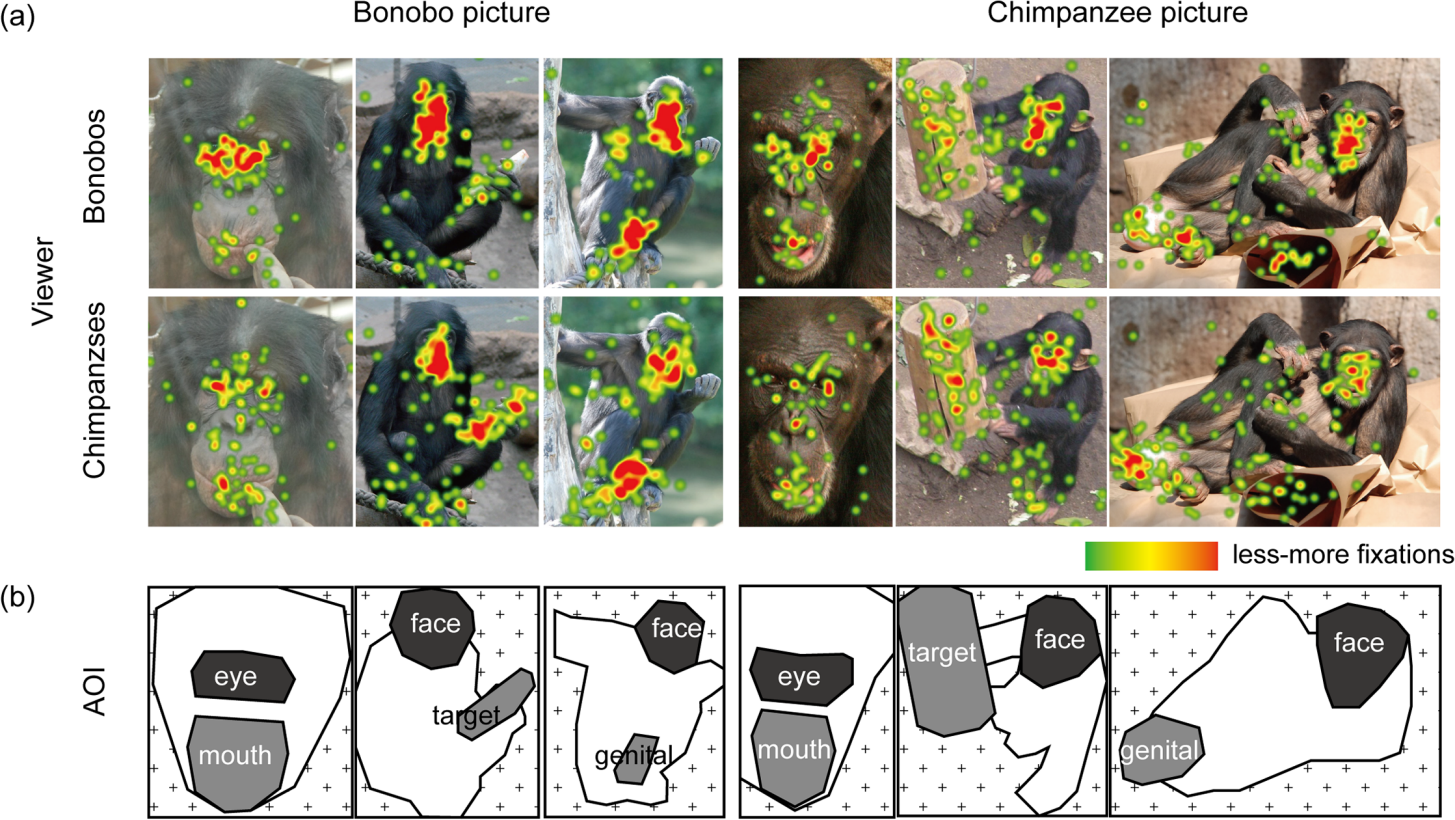
The examples for the viewing patterns by bonobos and chimpanzees, represented as the fixation heatmaps superimposed on the presented pictures. Each map was created from all fixations by all subjects that fell on the presented picture. In the map, the redder parts are more concentrated with fixations (each fixation had a Gaussian radius of 50 pixel). See Figure C in S1 File for the complete collection of heatmaps. (b) The examples for areas of interest (AOI).

AOIs (area of interest) were defined for the eyes and mouth in a full-face picture, and the face, target and ano-genital areas in a full-body picture, as shown in Fig 1. AOIs were marked without referencing the subjects’ viewing patterns. The viewing time to each AOI was then scored as the sum duration of fixations that fell within each AOI. Fixations were defined using the Tobii Fixation Filter in the Tobii Studio (version 3.2.1). Only fixations that begun 200 ms after the stimulus onset were included for the analysis because those fixations reliably reflect the responses to the presented stimuli.

To determine the factors influencing the viewing times to AOIs, we conducted a repeated-measures ANOVA (analysis of variance) with Subject species as the between-subject factor, and Model species and AOI as the within-subject factors. ANOVAs were conducted separately for the full-face trials (including the eye and mouth AOIs) and the full-body trials (including the face, target, and genital). These statistical analyses were conducted in SPSS 13.0. Additionally, to test the predictive value of overall viewing patterns for the species attributes, we conducted a discriminant analysis with the viewing times to AOIs (Eye, Mouth, Face (in a body), Target, and Genital) as continuous variables and species as a categorical variable.

## Results

Overall viewing patterns were highly similar between bonobos and chimpanzees. Bonobos and chimpanzees viewed the on-screen areas (vs. off-screen areas) for about the same amount of time (bonobo 2185 vs. chimpanzee 2303 ms; *t*(32) = 1.17, *p* = 0.24, Cohen’s d = 0.39). As expected, both species strongly viewed the main AOIs in the scenes; eyes, mouth, face, action target objects, and ano-genital parts, although bonobos and chimpanzees differed in the strength of viewing each AOI (Fig 1).

### Full-face trials

**Fig 2:**
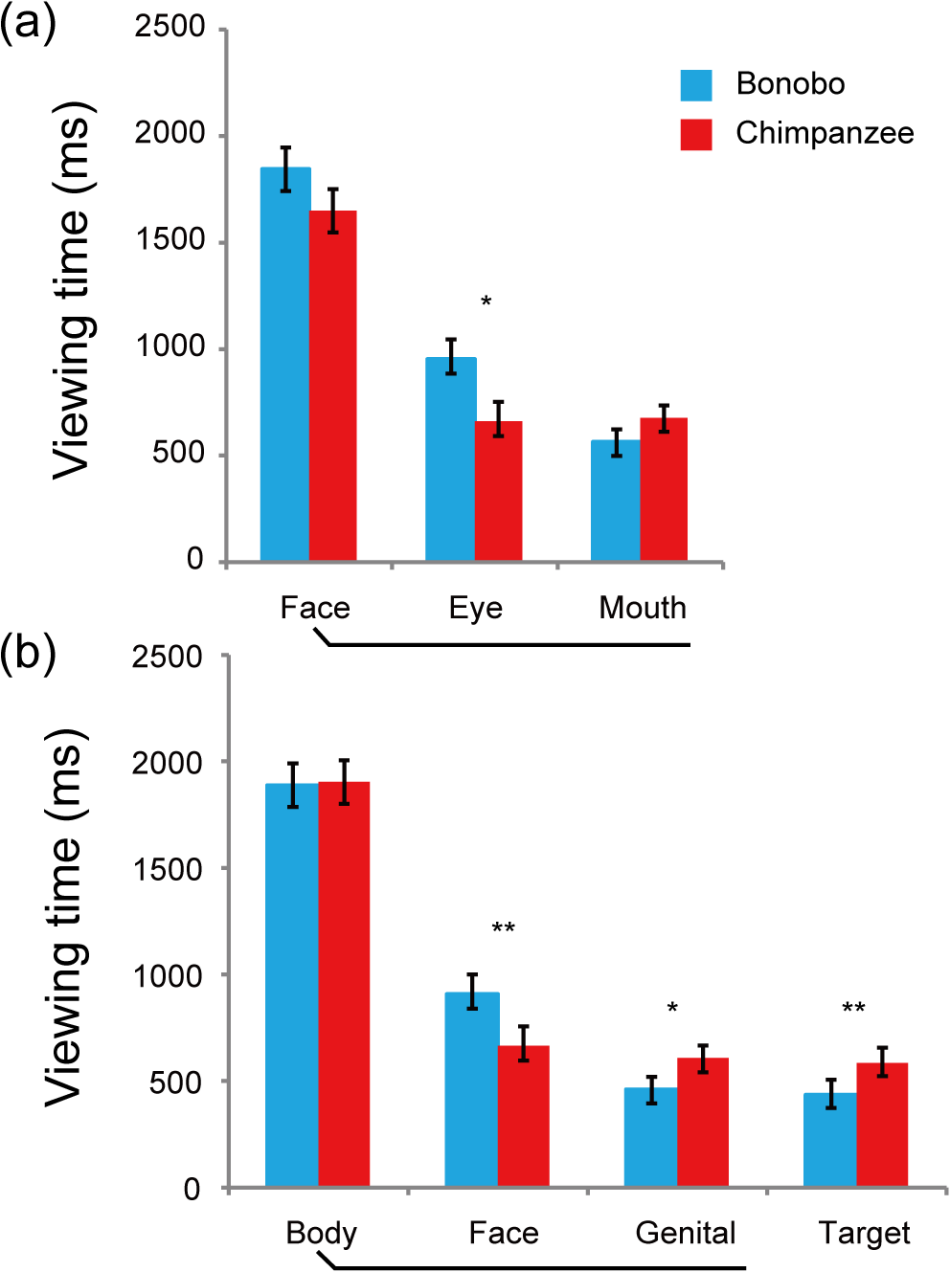
Viewing times (ms) for AOIs by bonobos and chimpanzees in the full-face trials (a) and the full-body trials (b). In the full-face trials, the face AOI included the eye and mouth AOI (and the others; ears, cheeks, forehead). In the full-body trials, the body AOI included the face and genital AOI (and the others; limbs and trunk). As bonobos and chimpanzees exhibited similar viewing patterns for the conspecific and allospecific pictures, the data is pooled over both species’ pictures (see Figure B in S1 File for the separated data). Error bars represent SEM.

Bonobos viewed the eyes rather than the mouth, while chimpanzees viewed the eyes and mouth for about the same durations (Fig 2A). A repeated-measures ANOVA with AOI (Eye and Mouth), Subject species (Bonobo, Chimpanzee) and Media species (Bonobo, Chimpanzee) as factors revealed the significant main effect of AOI (*F*(1,32) = 6.07, *p* = 0.019, partial *η* ^*2*^ = 0.15) in addition to a significant interaction between AOI and Subject species (*F*(1,32) = 7.19, *p* = 0.011, partial *η* ^*2*^ = 0.18). Post-hoc t-tests revealed that bonobos viewed the eyes significantly longer than chimpanzees (*t*(32) = 2.59, *p* = 0.014, Cohen’s d = 0.89). The observed species difference was independent of the presented species. That is, although both species altered their viewing patterns depending on the presented species (the main effect of Media species, *F*(1,32) = 13.56, *p* = 0.001, partial *η* ^*2*^ = 0.29; Media species×AOI, *F*(1,32) = 17.46, *p*<0.001, partial *η* ^*2*^ = 0.35), they did not show the differential patterns to the own vs. other species pictures (i.e. Media species×Subject species, n.s.; Media species×Subject species×AOI, n.s.). For the graphs of viewing patterns to each presented species, see Figure B in S1 File.

### Full-body trials

Bonobos viewed the face rather than the genitals or the action target objects, while chimpanzees viewed these AOIs for about the same durations (Fig 2B). A repeated-measures ANOVA with AOI (Face, Genital and Target), Subject species and Media species as factors revealed the significant main effects of AOI (*F*(2,64) = 24.41, *p*<0.001, partial *η* ^*2*^ = 0.43) in addition to a significant interaction between AOI and Subject species (*F*(2,64) = 12.95, *p*<0.001, partial *η* ^*2*^ = 0.28). Post-hoc t-tests revealed that bonobos viewed the face significantly longer than chimpanzees (*t*(32) = 3.51, *p* = 0.001, Cohen’s d = 1.22), while chimpanzees viewed the genitals and action target objects significantly longer than bonobos (*t*(32) = 2.06, 2.87, *p* = 0.047, 0.007, Cohen’s d = 0.74, 1.01, respectively). Bonobos viewed the face longer than chimpanzees even when we included the pictures that contained only the face and genitals (but not the target) (*t*(32) = 2.80, *p* = 0.009, Cohen’s d = 0.94) or only the face and the target (but not the genitals) (*t*(32) = 3.25, *p* = 0.003, Cohen’s d = 1.14).

As in the full-face trials, these observed species differences were independent of the presented species. That is, although both species altered their viewing patterns depending on the presented species (Media species×AOI; *F*(2,64) = 14.10, *p*<0.001, partial *η* ^*2*^ = 0.30), they did not show the differential patterns to the own vs. other species pictures (i.e. Media species×Subject species, n.s.; Media species×Subject species×AOI, n.s.). For the graphs of viewing patterns to each presented species, see Figure B in S1 File.

### Time course of viewing pattern within a trial

**Fig 3:**
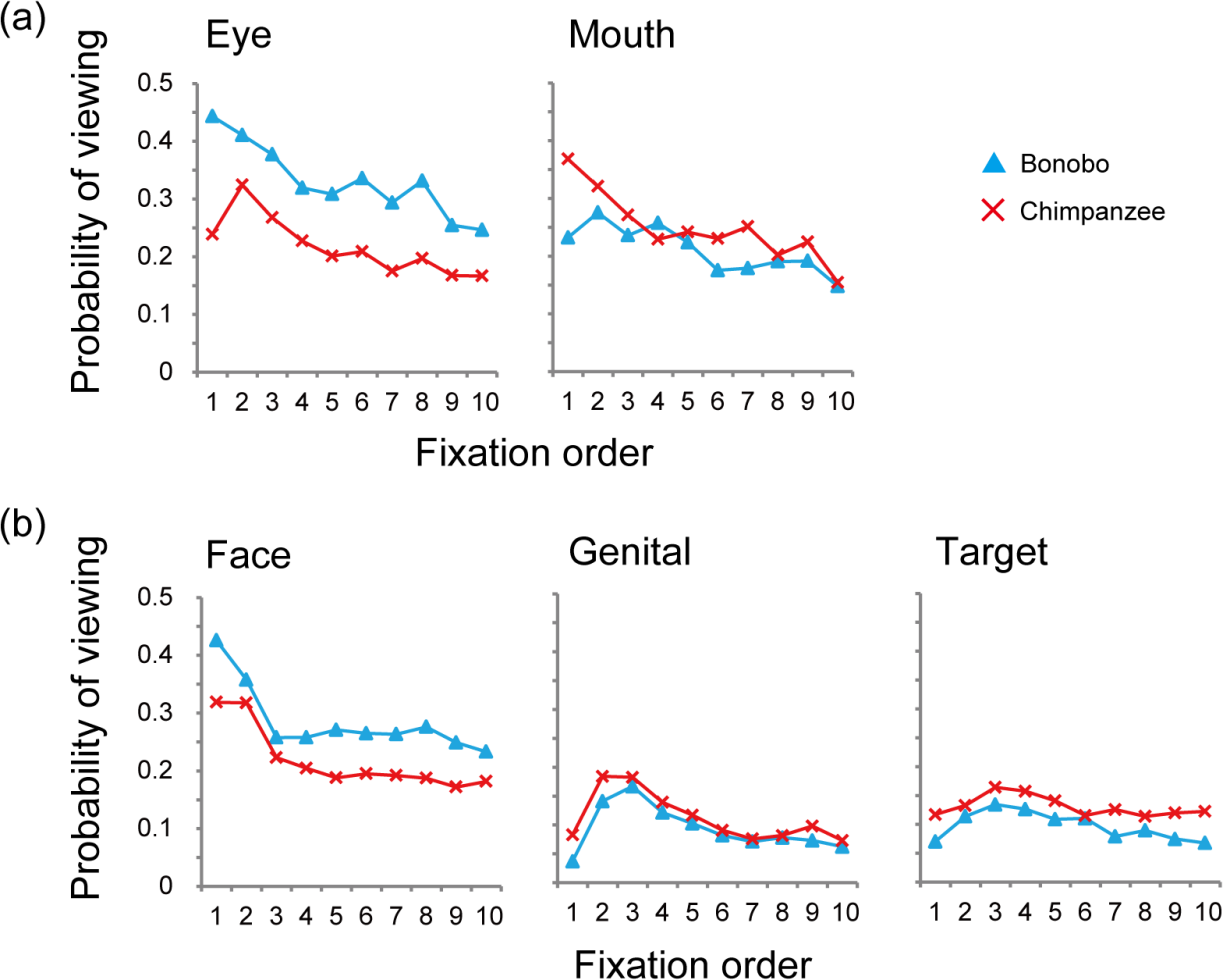
Time course of viewing patterns by bonobos and chimpanzees. The x-axis presents the fixation order, from the first to 10^th^ fixation timings, and the y-axis presents the probability of fixation (the number of trials in which the subjects fixated certain AOI) on each AOI. Error bars represent SEM.

Fig 3 shows the time course of viewing each AOI during the 3-sec. presentation time. The observed species differences were evident throughout the presentation time, even from the very first fixation. When presented with the full-face pictures, bonobos viewed the eyes rather than the mouth in the first fixation, while chimpanzees viewed the mouth rather than the eyes, showing the opposite patterns. When presented with the full-body pictures, both species viewed the face rather than the genitals or the action target objects in the first fixation, yet this tendency was stronger in bonobos than chimpanzees.

### Individual data and the discriminant analysis

Table 1 shows the individual data. The discriminant analysis revealed a single discriminant factor and the corresponding discriminant scores which showed the grouping patterns of the individuals (Table 1). This factor was most strongly correlated with the viewing time to the face, followed by the viewing time to the target, eyes, genitals, and mouth. Overall 85.3% of all individuals were correctly classified into their own species (misclassified: loto, yasa, lolita, ulla, riet). The conservative cross-validation (in which each case is classified by the functions derived from all cases other than that case) showed that overall 79.4% of all individuals were correctly classified (misclassified: loto, yasa, lolita, joey, ulla, riet, jamba). The inspection of miss-classified individuals did not reveal any common properties (e.g. living facility, sex, age class, rearing history) among these individuals; thus no confounding factor seem to be present in this classification based on the species attributes.

## Discussion

Bonobos viewed the face and eyes longer than chimpanzees. Instead of viewing the eyes, chimpanzees viewed the action target objects and ano-genital parts longer than bonobos. These species differences were partly due to a time trade-off, i.e. the longer viewing of faces led to the shorter viewing of the other attractive elements and *vice versa*. Yet, bonobos viewed the face longer than chimpanzees even though the pictures included the two different kinds of attractive elements, action target objects and ano-genital areas of other individuals, suggesting that bonobos actively maintained their attention to the face and eyes.

Bonobos fixated the eyes rapidly, even immediately after the picture presentation, and chimpanzees showed an opposite pattern; fixating the mouth rather than the eyes. In addition, bonobos viewed the eyes longer than chimpanzees independently of whether the presented stimulus was a conspecific face or an allospecific chimpanzee’s face. These results suggest that bonobos’ eye fixation was a well-automated response. A similar, rapid eye-fixation has been reported in humans [50, 51], from the early age [12], and also in several species of nonhuman primates (monkeys, gorillas, orangutans [16, 52]).

The viewing pattern of each individual robustly predicted the species in a discriminant analysis. It should be noted that, although we tested chimpanzees and bonobos from two separate facilities (WKPRC and KS), the results were highly consistent between the two facilities. This result eliminated the potential effect of some unavoidable procedural differences between facilities (see Method). Moreover, since our stimuli included models familiar to the WKPRC apes but not to the KS apes, the consistency of results between facilities indicated that model familiarity did not critically influence apes’ viewing patterns. In addition, as mentioned above, bonobos and chimpanzees showed similar fixation responses to the conspecific and allospecific face/eyes. Taken together these results suggest that the viewing pattern of each individual depended on a species-specific predisposition rather than on environmental or familiarity factors, at least in this experimental context (see [32, 53] for the other experimental context where the effects of model familiarity were observed in great apes). In humans, the individual variation of eye contact is also generally stable across contexts and different counterparts [8].

We also confirmed our prediction that chimpanzees pay more attention to action target objects than bonobos. This attentional difference may be related to the cognitive differences between chimpanzees and bonobos [31]. In particular, bonobos’ increased performance in the test related to theory-of-mind, especially gaze-following, may partly depend on their increased attention to the experimenter’s face and eyes. Also, chimpanzees’ increased performance in the test requiring tool-using or an understanding of physical causality may partly depend on their increased attention to the experimenter’ action and the target objects, relative to bonobos (or the bonobos’ inattentiveness to the action target objects). Although previous studies generally support the idea that overt attention influences subsequent behaviors in nonhuman primates [54–56], the increased attention to the action target objects or the eyes may not directly influence their performance in every task. For example, in a behavioral task requiring an inhibitory control of overt looking behavior for deceptive purposes in a competitive task, the individuals should not follow such general pattern. Future studies are needed to test this idea.

Alternatively, those attentional differences between species in this study (i.e. free viewing without any task demand) may indicate differences in their [57][54][55]motivation or “interest” level, which may then influence the cognitive performance in previous studies. It should also be noted that the increased attention to the action target objects by chimpanzees relative to bonobos may be partly due to the time trade-off, as mentioned above (i.e. due to the decreased attention to the face). Thus, to better confirm the idea that chimpanzees are differently motivated from bonobos to explore the action target objects, we should gather further evidence from multiple contexts.

The data from this and other studies suggest that there may be common neural, hormonal, and genetic mechanisms underlying eye contact and affiliation in human and nonhuman primates. As mentioned above, eye-fixation differences between bonobos and chimpanzees in this study may resemble the differences that have been previously reported between TD and ASD infants using the same eye-tracking method [28]. In a recent study, consistent with human data [58], oxytocin-administered macaques showed an increased attention to the eyes of conspecific images after oxytocin administration [59]. Relatedly, the oxytocin-receptor gene is reported to be different between bonobos and chimpanzees [60]. Also see recent studies that showed the human-dog bonding and communication mediated by eye-contact, human social cues, and oxytocin [61, 62]. In humans, individuals with a higher level of prenatal androgens show a decreased level of eye contact [63]. Relatedly, prenatal androgens are hypothesized to be higher in chimpanzees than in bonobos, as suggested by their differences in 2D-4D (digit) ratio [64]. Also, consistent with human data [65, 66], the neurons in the monkeys’ amygdala responded to the eyes selectively when they were fixating on the conspecific eyes in the video scenes [67]. One study comparing the local gray matter between bonobos and chimpanzees found differences in the regions involved in the brain network related to social communication [68], which, in humans, is activated when making eye contact [27]. Further cross-species studies focusing on the neural and cognitive mechanisms of social attention should enhance our understanding of the evolutionary origin of eye contact and the basic social motivation underlying complex social behaviors and cognition.

In conclusion, we observed differences between bonobos and chimpanzees in their attention to social and physical elements. We suggest that, if such attentional or motivational differences have emerged between bonobos and chimpanzees in a relatively short period of time (1–2 million years), those changes could have influenced the development and evolution of behaviors and cognition of these species in important ways. Finally, just as it may have happened to *Pan* species, the evolutionary shift in attentional and motivational systems may have partly contributed to shaping the species-unique behaviors and cognition of humans even in a relatively short period of evolutionary time.

## Supporting Information

S1 File Supporting file that also contains Figures A-C. Figure A shows the eye-tracking setting with chimpanzees and bonobos. Figure B shows the viewing times (ms) for AOIs in the bonobo and chimpanzee pictures by bonobos and chimpanzees. Figure C shows a complete collection of fixation heatmaps superimposed on the presented pictures.
(DOCX)

S1 Video Examples of viewing patterns by a bonobo Vijay and a chimpanzee Natsuki. The lines indicate saccades and the circles indicate fixations (scaled to the durations).
(MP4)
