# Correction: Social Attention in the Two Species of Pan: Bonobos Make More Eye Contact than Chimpanzees

**DOI:** 10.1371/journal.pone.0133573

**Published:** 2015-07-15

**Authors:** The PLOS ONE Staff

An affiliation for the first author is missing. Fumihiro Kano is also affiliated with Kumamoto Sanctuary, Wildlife Research Center, Kyoto University, Kumamoto, Uki, Japan. The publisher apologizes for this error.

## References

[1] Kano F, Hirata S, Call J (2015) Social Attention in the Two Species of Pan: Bonobos Make More Eye Contact than Chimpanzees. PLoS ONE 10(6): e0129684 doi:10.1371/journal.pone.0129684 26075710 10.1371/journal.pone.0129684 PMC4468221

