# Supplementary material for: Social Attention in the Two Species of Pan: Bonobos Make More Eye Contact than Chimpanzees
Source: PLoS One. 2015 Jun 15;10(6):e0129684. doi: 10.1371/journal.pone.0129684 (PMC4468221; doi:10.1371/journal.pone.0129684)
Supplement: S1 File — Figure A shows the eye-tracking setting with chimpanzees and bonobos. Figure B shows the viewing times (ms) for AOIs in the bonobo and chimpanzee pictures by bonobos and chimpanzees. Figure C shows a complete collection of fixation heatmaps superimposed on the presented pictures. (DOCX) [file pone.0129684.s001.docx]

**S1 File**

**Ethics statement**

In both facilities, the living areas were large and complex enough for the apes to rest, exercise, and socialize with the group mates. The outdoor playground areas were larger than 200 m^2^ and were equipped with climbing trees, vegetation and enrichment devices. The indoor areas including sleeping rooms were larger than 100 m^2^. The apes received fresh fruits, vegetables, nuts and leaves distributed in three main meals and occasional enrichment programs. Water was available ad libitum throughout the day. They voluntarily participated in the study and were never food or water deprived. In KS, the bonobos were tested in one of their sleeping rooms (15 m^2^), and the chimpanzees were tested in a separate testing room (9 m^2^). In WKPRC, all apes were tested in one of their sleeping rooms (9 m^2^). The research was noninvasive and no medical, toxicological or neurobiological research of any kind is conducted at KS [[1](#_ENREF_1)] or WKPRC.

Animal husbandry and research complied with the international standards in accordance with the recommendation of the Weatherall report “The use of non-human primates in research” and the local guidelines which are strictly adhered to the national laws of Japan or Germany [KS: Primate Research Institute “Guide for the Care and Use of Laboratory Primates 3rd Edition”, Wildlife Research Center “Guide for the Animal Research Ethics”] [WKPRC: “EAZA Minimum Standards for the Accommodation and Care of Animals in Zoos and Aquaria”, “WAZA Ethical Guidelines for the Conduct of Research on Animals by Zoos and Aquariums”, “Guidelines for the Treatment of Animals in Behavioral Research and Teaching” of the Association for the Study of Animal Behavior (ASAB)]. The study protocol was approved by the institutional committee of Wildlife Research Center (No. WRC-2014KS001A) and Max-Planck Institute for Evolutionary Anthropology.


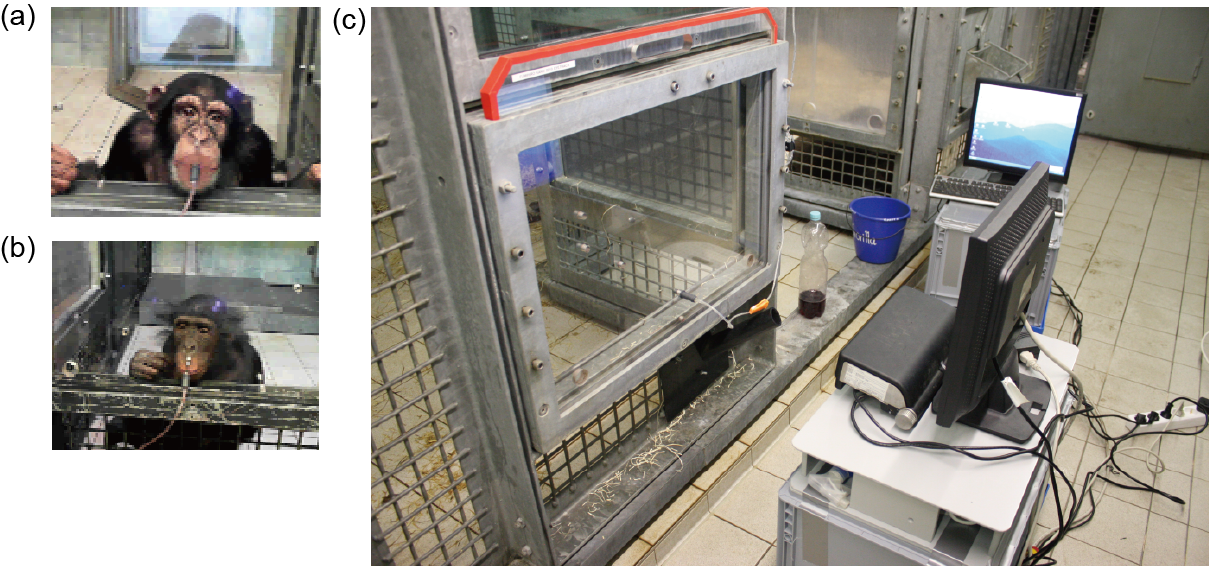


Figure A. A chimpanzee (a) and a bonobo (b) on the eye-tracking setting (c). The eye-tracker is set below the monitor.

**
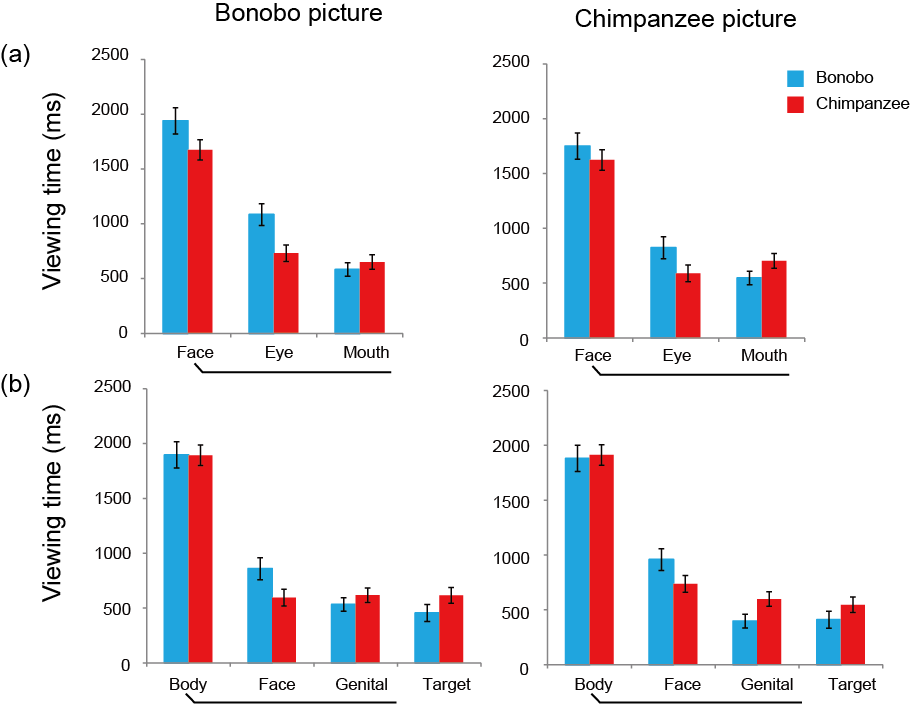
**

Figure B. Viewing times (ms) for AOIs in the bonobo and chimpanzee pictures by bonobos and chimpanzees.

**
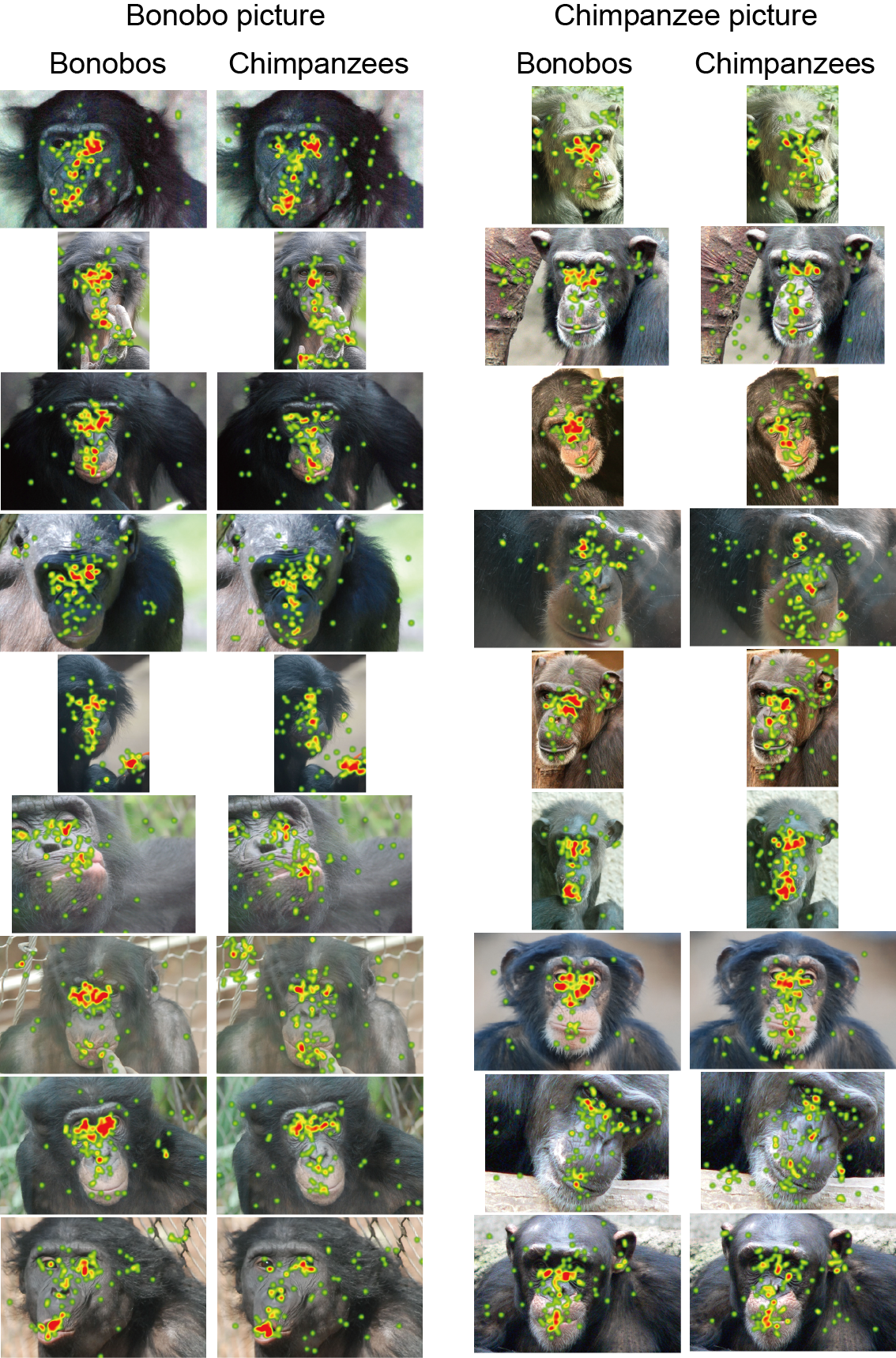
**

**
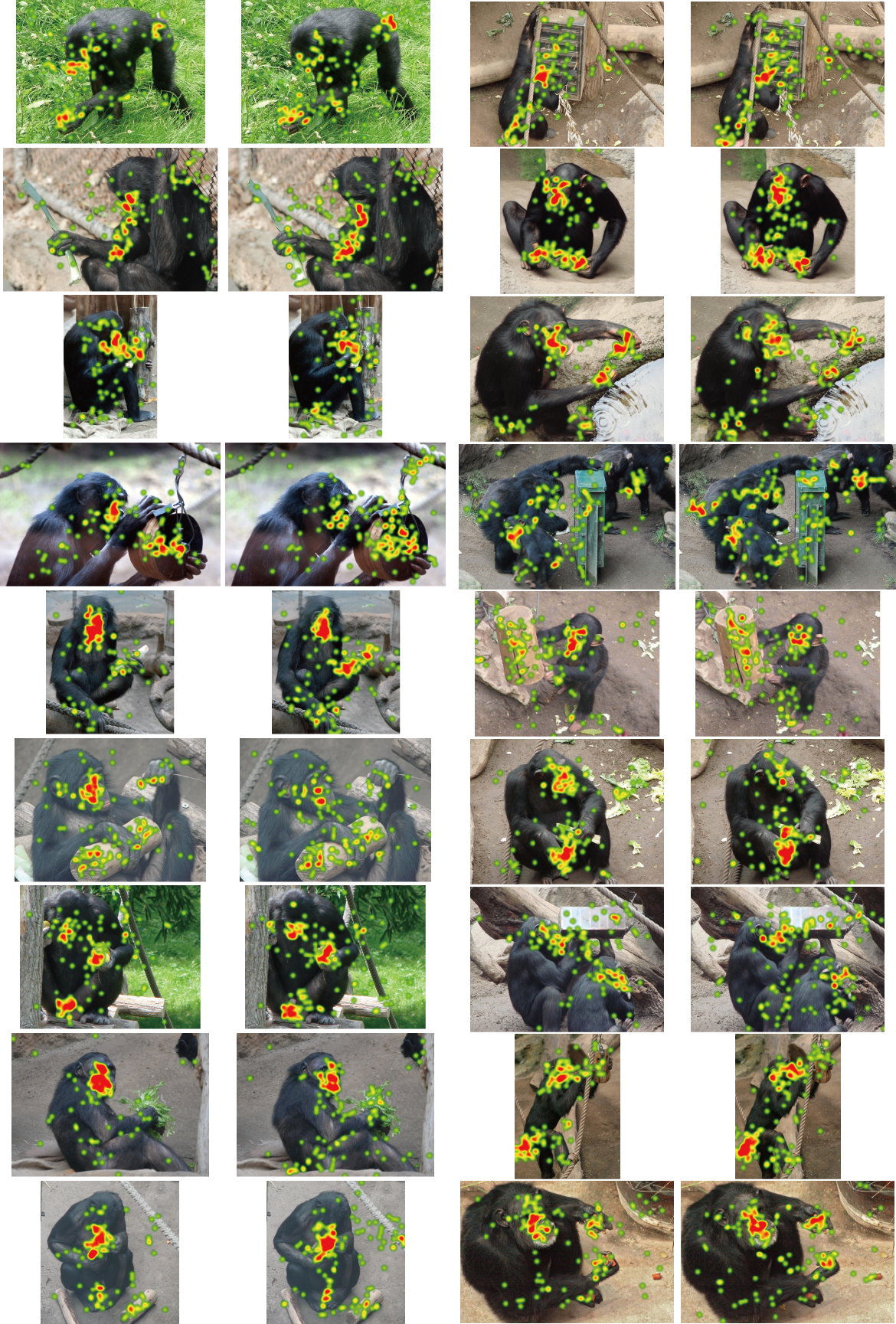
** **
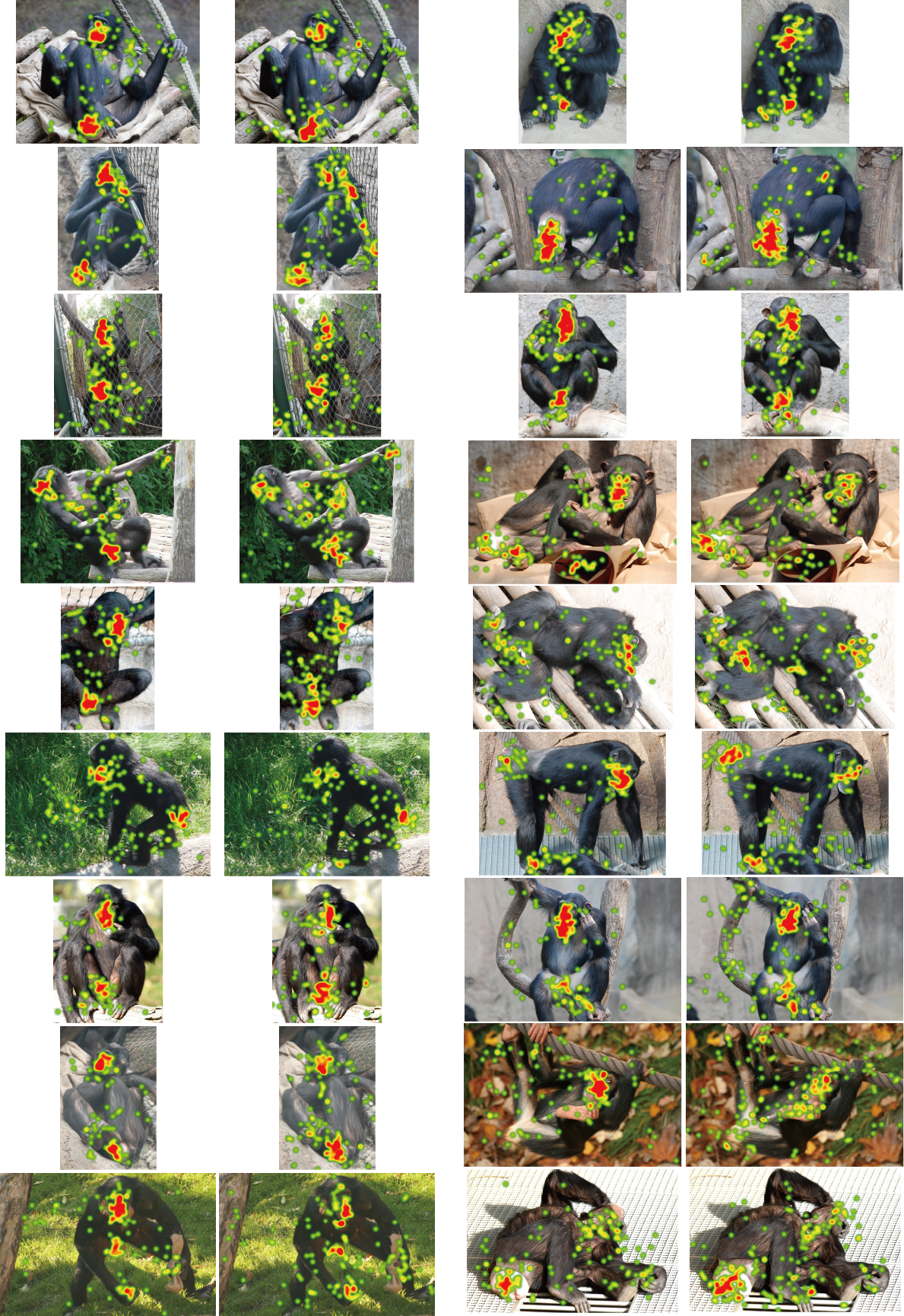
**

**
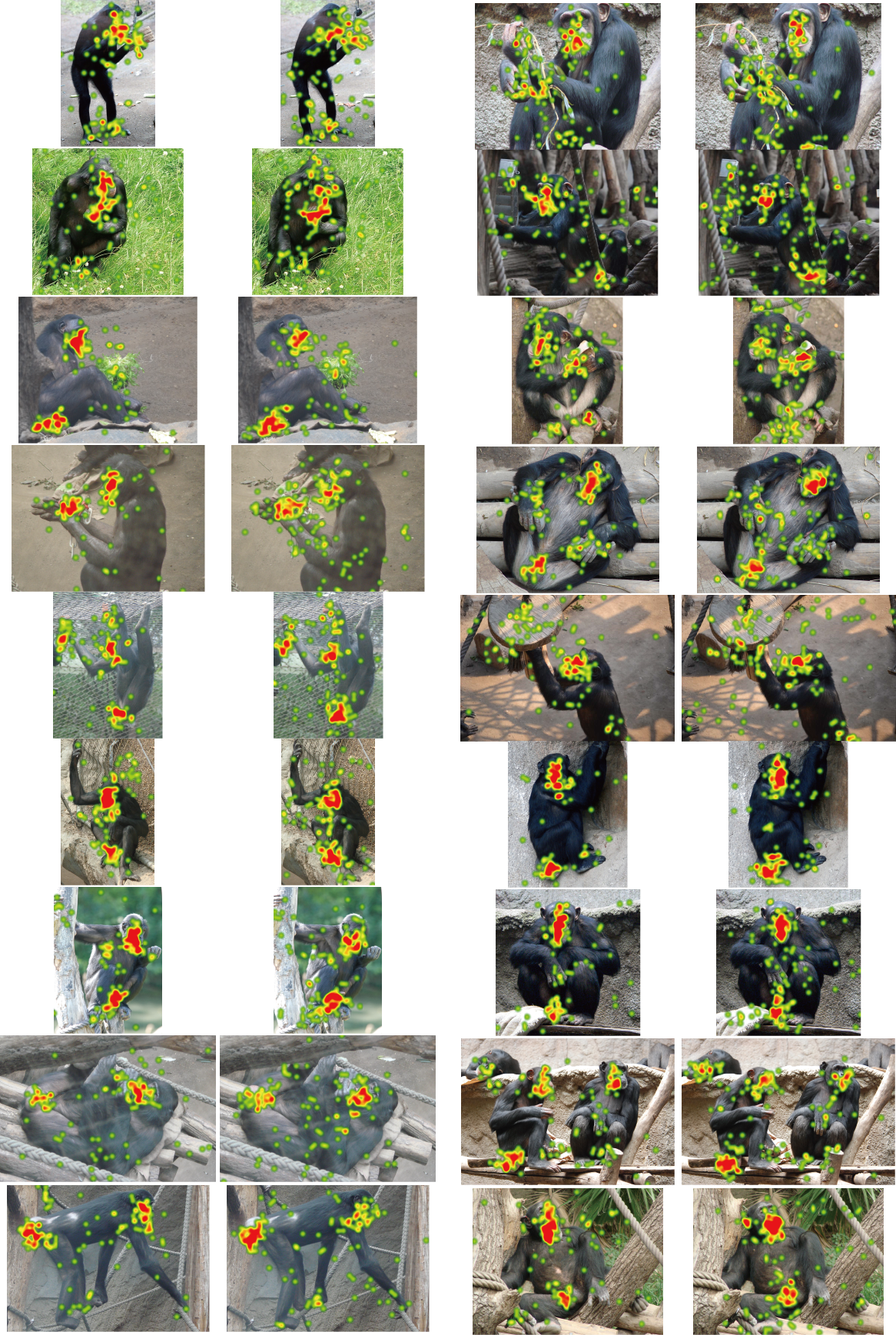
**

**
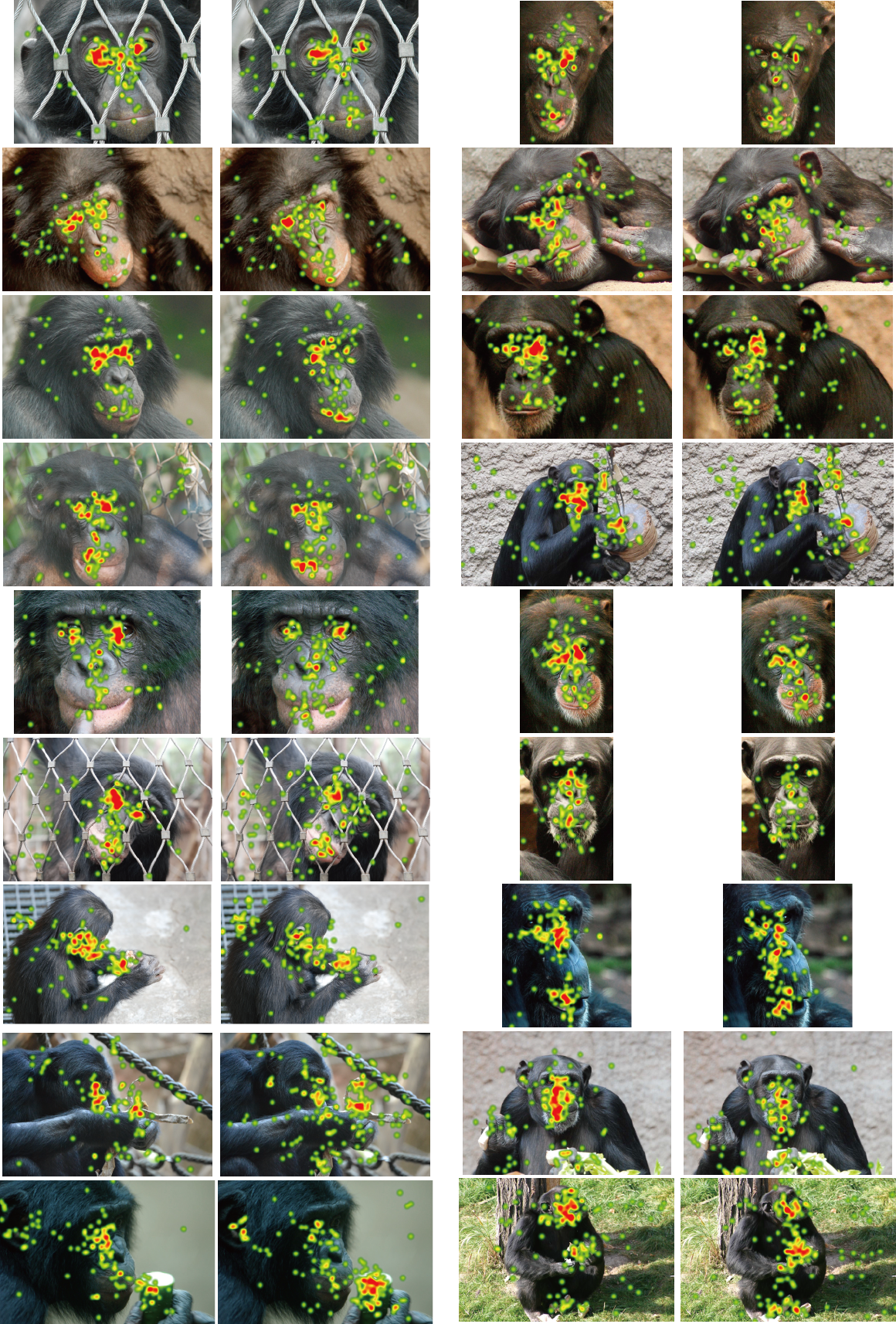
**

Figure C. A complete collection of fixation heatmaps superimposed on the presented pictures. The left heatmap is bonobos’ and the right heatmap is chimpanzees’. The left two pictures are the bonobo models, and the right two pictures are the chimpanzee models.

1. Morimura N, Idani G, Matsuzawa T (2011) The first chimpanzee sanctuary in Japan: an attempt to care for the “surplus” of biomedical research. American journal of primatology 73: 226-232.
